# Health and Economic Benefits of Improved Injury Prevention and Trauma Care Worldwide

**DOI:** 10.1371/journal.pone.0091862

**Published:** 2014-03-13

**Authors:** Meera Kotagal, Kiran J. Agarwal-Harding, Charles Mock, Robert Quansah, Carlos Arreola-Risa, John G. Meara

**Affiliations:** 1 Department of Surgery, University of Washington, Program in Global Surgery and Social Change, Harvard Medical School, Seattle, Washington, United States of America; 2 Department of Plastic and Oral Surgery, Boston Children’s Hospital, Program in Global Surgery and Social Change, Harvard Medical School, Boston, Massachusetts, United States of America; 3 Harborview Injury Prevention and Research Center, Harborview Medical Center, University of Washington, Seattle, Washington, United States of America; 4 Department of Surgery, School of Medical Sciences, Kwame Nkrumah University of Science and Technology, Kumasi, Ghana; 5 Department of Emergency Medicine, School of Medicine, Tecnologico de Monterrey, Monterrey, Mexico; 6 Department of Plastic and Oral Surgery, Boston Children’s Hospital, Program in Global Surgery and Social Change, Harvard Medical School, Boston, Massachusetts, United States of America; Monash University, Australia

## Abstract

**Objectives:**

Injury is a significant source of morbidity and mortality worldwide, and often disproportionately affects younger, more productive members of society. While many have made the case for improved injury prevention and trauma care, health system development in low- and middle-income countries is often limited by resources. This study aims to determine the economic benefit of improved injury prevention and trauma care in low- and middle-income countries.

**Methods:**

This study uses existing data on injury mortality worldwide from the 2010 Global Burden of Disease Study to estimate the number of lives that could be saved if injury mortality rates in low- and middle-income countries could be reduced to rates in high-income countries. Using economic modeling – through the human capital approach and the value of a statistical life approach – the study then demonstrates the associated economic benefit of these lives saved.

**Results:**

88 percent of injury-related deaths occur in low- and middle-income countries. If injury mortality rates in low- and middle-income countries were reduced to rates in high-income countries, 2,117,500 lives could be saved per year. This would result in between 49 million and 52 million disability adjusted life years averted per year, with discounting and age weighting. Using the human capital approach, the associated economic benefit of reducing mortality rates ranges from $245 to $261 billion with discounting and age weighting. Using the value of a statistical life approach, the benefit is between 758 and 786 billion dollars per year.

**Conclusions:**

Reducing injury mortality in low- and middle-income countries could save over 2 million lives per year and provide significant economic benefit globally. Further investments in trauma care and injury prevention are needed.

## Introduction

Injury is a significant source of morbidity and mortality worldwide, particularly in low- and middle-income countries (LMICs). There are over four million injury-related deaths yearly, more than the number of deaths from HIV/AIDS, malaria and tuberculosis combined [1]–[3]. Young people, often the most productive members of society, are at particular risk with injuries causing over 40% of their deaths [4]. The Global Burden of Disease (GBD) Study 2010 found that injuries caused 11.2% of all disability-adjusted life-years (DALYs) worldwide, and that, of these, 27% are from road injuries alone [5]. Road injuries are the eighth leading cause of mortality worldwide, accounting for 53% more deaths than tuberculosis [1], [5], [6].

Mock et al. estimated that between 1,730,000 and 1,965,000 lives could be saved worldwide if case fatality rates among seriously injured persons in LMICs could be reduced to rates of high-income countries (HICs) through improvements in trauma care (care of the injured). This would avoid 34–38% of all current injury-related deaths [7]. However, this study was limited by the scarcity of data on LMIC injury incidence and mortality, and extrapolated rates for LMICs from three cities (Kumasi, Ghana, Monterrey, Mexico, and Seattle, USA) representing low-, middle-, and high-income countries respectively. Additionally, while examining the numbers of lives lost is valuable in making a case for increased investment in trauma care, health system development in LMICs is often limited by resources. It is therefore valuable to quantify the economic benefit of reducing mortality, through both injury prevention and improved trauma care. Several studies have shown, with economic modeling and cost effective analysis, that surgery is cost effective and can provide tremendous economic benefit [8]–[13].

The 2010 GBD study, with country-specific values of injury-related deaths, years of life lost (YLLs), and disability-adjusted life-years (DALYs) for over 190 countries, makes it possible to examine injury mortality in greater detail, as well as to estimate the economic benefit of injury mortality reduction. In this study, using data from the 2010 GBD study, we aim to quantify the number of lives that could be saved worldwide, as well as the resulting potential economic benefit.

## Methods

### Study Data

To model the health and economic effects of reducing injury mortality, we used data from the World Bank and the 2010 GBD study [1], [5], [14]. All countries with published estimates for total population, life expectancy, and gross national income per capita (GNIpc) by both the Atlas and Purchasing Power Parity (PPP) method were included in the study (166 countries) [14]. We used World Bank income definitions to separate countries into low-income (32 countries), lower middle-income (46 countries), upper middle-income (44 countries), and high-income (44 countries). These categories represent the income group of the country based on per capita income, and the entire population of each country is counted within that country income group, regardless of any one individual’s income. The total population included in this study was 6.64 billion, approximately 95% of the estimated 6.97 billion people worldwide [14].

### Ethics Statement

Ethics committee approval was not required for this study as the study used publicly available, country-level, de-identified data.

### Characterizing the Burden of Trauma-related Deaths

The 2010 GBD study provides up-to-date, country-by-country estimates of total deaths, YLLs, and DALYs for twelve mechanisms of injury (road injuries, interpersonal violence, mechanical forces, drowning, poisonings, fire, self-harm, falls, other transport injuries, animal contact, war and legal intervention, and forces of nature), and sixteen age groups (1–4 years old, 5–9 years old, 10–14 years old, 15–19 years old, 20–24 years old, 25–29 years old, 30–34 years old, 35–39 years old, 40–44 years old, 45–49 years old, 50–54 years old, 55–59 years old, 60–64 years old, 65–69 years old, 70–74 years old, 75–79 years old, 80 years and older) [1], [5]. For each mechanism of injury, we calculated the number and percentage of all injury deaths by country income group. Next, for each country income group, we calculated the number and percentage of total deaths from each mechanism of injury.

We then summed total deaths (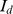), YLLs, and DALYs from all mechanisms of injury for each country by age group and overall, and subsequently for all countries in each country income group. Since DALYs are the sum of YLLs and YLDs, we divided total YLLs by total DALYs in each income group to calculate the percentage of DALYs due to YLLs. This helped elucidate the extent to which DALYs were secondary to mortality rather than disability. To estimate YLDs, we subtracted YLLs from DALYs for each age group and country. Additionally, we calculated YLLs and DALYs with discounting and age weighting. Discounting the value of future DALYs to their present value is commonly performed in order to improve economic comparability of DALYs that occur at different points of time. Consistent with prior studies, we applied a discount rate of 3% in our calculations of YLLs [8], [9], [10], [15]. According to the 2010 GBD, YLDs were calculated as prevalence of a sequela multiplied by the disability weight for that sequela [6]. Therefore, YLDs are experienced only at the present time, unlike YLLs which are based on incidence and years of future life that are lost, so we performed discounting on YLLs but not on DALYs. However, age weighting of present value YLLs and YLDs was performed. The common justification for age weighting DALYs is that the social and economic value of a year of healthy life is greater for young adults than for young children or the elderly. The age at which the DALY function peaks is determined by the parameter β, with the peak occurring at 1/β. A value of 0.04 is commonly used for β corresponding to a peak at age 25 [15]. We performed age weighting of YLLs and YLDs using this value for β, as well as an additional value of 0.017 (denoted here as 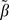 based on a peak occurring at two-thirds of life expectancy at birth, which has been shown to be more consistent with empirical evidence on valuation of health risks [8], [16]. Per the 2010 GBD methodology, a standardized life expectancy table was used, with life expectancy at birth of 86.0 years [4], [17]. Like previous studies, we adopted the following notation to indicate our age weighting and discounting parameters used in calculating YLLs, YLDs, and DALYs: DALYs [r,K,β], where r = the discount rate, K = modulation of age-weighting formula (0 = age weights off, 1 = age weights on), and β = age weighting parameter. For example, DALYs [3,1,β] indicates a 3% discount rate and age weighting, with a β value of 0.04 [2]–[4]. In addition to YLLs [0,0,0] provided by the 2010 GBD study, we calculated YLLs [3,1,β] and YLLs [3,1,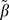] using the total deaths by age group for each country, and the standardized life expectancy by age group. We used the 2010 GBD values of YLDs [0,0,0] by age group to calculate YLDs [0,1,β] and YLDs [0,1,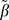]. In addition to the 2010 GBD study’s values of DALYs [0,0,0], we calculated DALYs [3,1,β] and DALYs [3,1,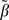] by summing YLLs [3,1,β] and YLDs [0,1,β], and summing YLLs [3,1,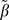] and YLDs [0,1,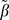], respectively.

Using the total population of each country income group, by age group, we calculated the rates (number per 100,000 people) for deaths (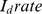), and DALYs (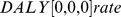, 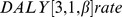, 
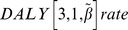
).

### Estimating the Number of Deaths Prevented

To calculate the lives saved by reducing injury mortality in LMICs, we first calculated a new theoretical incidence of injury deaths (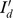) by age group for each country if the injury-related death rate in each age group were equal to the corresponding age-group-specific rate in HICs:
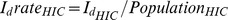





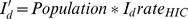
where 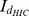 and 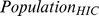 are total injury-related deaths and total population in HICs, respectively, from a specific age group. We calculated 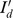 by age group for each country and summed them by age group and by country income group. For each country, we subtracted 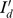 from 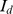 to yield the net lives saved per country by age group and overall, which we then summed by country income group and overall.

### Estimating DALYs Prevented

DALYs are a health metric used to quantify the combined burden of mortality and morbidity. DALYs - the sum of YLLs and YLDs - reflect the incidence of injury, rates of injury-related death and disability, age of injury, average healthy life expectancy, and disability severity. These factors are inherently linked since public health interventions targeting one factor would affect them all to varying degrees. Therefore, these factors must all be considered when estimating DALYs averted by reducing injury mortality.

In the theoretical situation where injury mortality rates in LMICs match those of HICs, this would be achieved with system-wide improvements in both injury prevention and trauma care. The magnitude of the effect of these interventions in LMICs on the factors involved in DALY calculations likely varies on a continuum between these factors remaining constant at LMIC levels or approximately matching HIC levels. We therefore calculated a range of estimates for net DALYs averted, by subtracting the actual DALYs in LMICs from the theoretical new DALYs if conditions (including both injury rates and injury mortality rates) in LMICs matched HICs. At one extreme, we calculated the theoretical DALYs if all factors were held constant at LMICs rates except for injury-related death rates (*method 1*). At the other extreme, we calculated the theoretical DALYs with all factors matching conditions in HICs (*method 2*). Unfortunately, we were unable to control for population injury rates since the 2010 GBD study data provided injury-related death incidence, but not injury incidence. We used population injury death rates (deaths per 100,000 people), which combine population injury rates (injuries per 100,000 people) and injury mortality (deaths per 100,000 injuries).

For *method 1*, we assumed that injury-related death rates alone would match rates in HICs. Assuming disability rates would not change, YLDs would be constant and cancel out in the net DALY calculation. Therefore, we only calculated YLLs. YLLs due to injury-related deaths for a LMIC, Country X, are calculated by age group as follows:
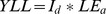
where 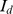 is the incidence of injury-related deaths in a specific age group, and 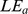 is the average life expectancy of that age group and thus the average number of years lost per injury-related death.

Using 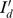 as the new incidence of injury mortality if mortality rates are reduced to the average rate in HICs (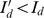) and the standardized life expectancy tables from the 2010 GBD study, we calculated new estimates of YLL by age group for each country, with and without age weighting and discounting (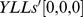, 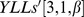, and 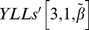).

Here we assume that YLL changes in isolation, without changes in YLD. In other words, the same people are getting the same injuries, but those people are dying from their injuries at a reduced rate with no change in the disability rates. However, as people avoid death, some will live with disabilities, causing a shift in DALYs from YLLs to YLDs. Therefore, in *method 2*, we calculated 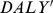 as the DALYs for Country X if injury incidence, mortality, life expectancy, age of injury, and disability rates were altered to match those of HICs.

Using country-specific estimates for DALYs from the 2010 GBD study [18] and our age-weighted and discounted estimates of DALYs, 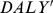 was calculated by age group as follows:
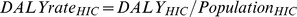





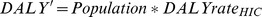



We calculated new estimates of DALYs by age group for each country, with and without age weighting and discounting (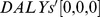, 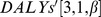, and 
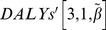
).

We calculated 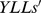 and 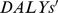, with and without age weighting and discounting, for each country by age group and summed them by age group and by country income group. For each country, if 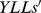
was less than 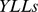, or 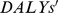 was less than 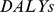 we calculated the net DALYs averted as follows:
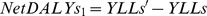





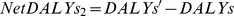



We summed 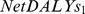 (*method 1*) and 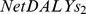 (*method 2*), respectively, for each country income group and overall. This was performed separately for each of our three age weighting and discounting conditions ( [0,0,0], [3,1,β], and [3,1,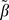]).

### Estimating the Economic Benefit of Trauma Mortality Reduction

Similar to previous studies, we used two methods to translate DALYs to U.S. dollars in our economic model: the human capital approach and the value of a statistical life (VSL) approach. The human capital approach implies that every individual is worth what he can contribute to his national economy [19]. DALYs incurred by disease are years detracting from this productivity, so DALYs are therefore valuated by the gross national income per capita (GNIpc). As with previous studies, we used the purchasing power parity (PPP) method, instead of the Atlas method, for calculating GNIpc because PPP better accounts for differences in relative price levels across countries and is thus a more valid cross-country measure of income per capita [8]–[10], [20] GNIpc is an estimate of the average individual’s productivity for a specific country, recognizing that any given individual may contribute more or less than the gross national income (GNI) of their country. For every country, we calculated the economic benefit (human capital approach) as follows:
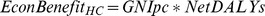
where 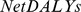 for a given country are the total net DALYs across all age groups. As with prior studies, we calculated 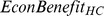 using DALYs with and without discounting and age weighting with a peak age of 25 (β = 0.04) [8], [9]. Four estimates of 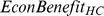 were generated for each country, using 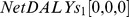, 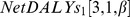, 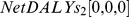, and 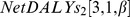.

For the VSL approach, we applied the concept that VSL is based on how much a person is willing to pay to avoid an undesirable outcome, e.g. premature death. This is determined using studies that measure willingness to pay or empirical wage data [20] and forms the basis of cost-benefit analyses globally [21]. The VSL approach may be preferable to the human capital approach as it is more grounded in economic theory and empirical study of human behavior [8]. The Environmental Protection Agency estimates the VSL in the United States as $7.4 million [22]. This value was adjusted for Country X as follows:
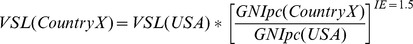



IE is the income elasticity coefficient of 1.5 [10]. Country-specific estimates of VSL were converted to annualized equivalents (VSLY) by treating VSL as the present value of an annuity with VSLY being the constant annual payment over x years of remaining life [21].

For every country, we calculated the economic benefit (VSL approach) by multiplying the country-specific valuation term VSLY by the net DALYs averted:
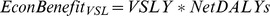



As with prior studies, for consistency with our calculation of the VSLY term, 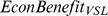
was calculated only using net DALYs with discounting and age weighting to two-thirds of life expectancy (β = 0.017) [8], [9], [10]. Two estimates of 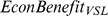 were generated using 
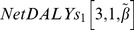
 and 
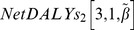
, respectively. For each country income group and overall, we calculated the sum of the economic benefit from both approaches.

## Results

### Overall Deaths and Years of Life Lost

Eighty-four percent of the world lives in LMICs - 11% in low-income, 37% in lower middle-income, 36% in upper middle-income, and 16% in high-income countries. From the 166 countries and 16 age groups included in this study, there are 4,389,560 injury-related deaths yearly. The injury-related death rate (per 100,000 people) was 90.4 in low-income countries, 72.5 in lower middle-income countries, 60.9 in upper middle-income countries, and 47.5 in HICs. The worldwide death rate was 66.1. Globally, without age weighting or discounting as is the standard of the 2010 GBD study, over 237 million DALYs are lost due to injury, 197 million of which are YLLs (table 1).

**Table 1:** Totals and rates of death, YLLs, and DALYs by income group.

Totals and rates of death, YLLs, and DALYs by income group			
IncomeGroup	Total deaths, in thousands(overall death rate, per 100,000 people)	Total YLLs, in thousands (overallYLL rate, per 100,000 people)	Total DALYs, in thousands (overallDALY rate, per 100,000 people)
Low	637 (90.4)	33,843 (4,806)	36,884 (5,238)
Lower Middle	1,782 (72.5)	86,483 (3,517)	99696 (4,055)
Upper Middle	1,452 (60.9)	61,085 (2,561)	75480 (3,164)
LMICs	3,871 (69.8)	181,411 (3,270)	212,060 (3,822)
High	518 (47.5)	16,174 (1,482)	25,307 (2,318)
World	4,390 (66.1)	197,585 (2,976)	237,367 (3,575)

Totals are aggregated from all twelve mechanisms of injury and all countries in each income group. Rates are calculated as the total deaths, YLLs, or DALYs divided by total population of each income group. In accordance with the standard of the 2010 GBD study, values are presented here without discounting or age weighting.

### Causes of Death

**Figure 1:**
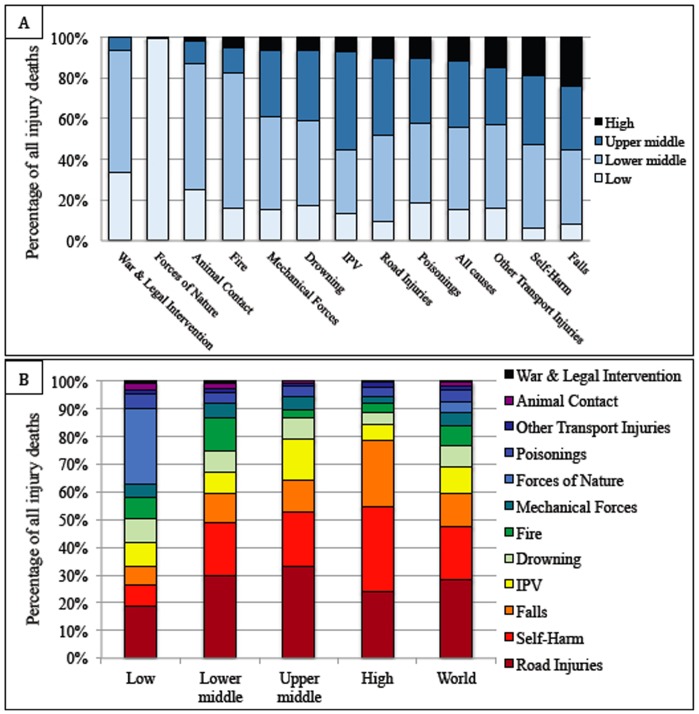
Percentage of total injury deaths by income level and injury type. **A)** Percentage of total injury deaths by income level for each mechanism of injury. The mechanisms of injury are sorted on the x-axis from greatest-to-least by the percentage of deaths occurring in LMICs. **B)** Percentage of total injury deaths by injury type in each income group. The mechanisms of injury are sorted in the columns from greatest-to-least by the percentage of total deaths occurring worldwide.

The vast majority of injury-related deaths (88%) occur in LMICs. The prevalence of individual causes of death varies by income group. Self-harm and falls were more common causes in HICs (30 and 24%, respectively) than in low- (8 and 7%, respectively) and middle- (20 and 11%, respectively) income countries (Figure 1). Conversely, deaths due to war and legal intervention and forces of nature were more common in LMICs. Globally, road injuries remain a significant source of mortality, causing 28% of injury deaths. This substantial impact is true across income groups, ranging from 19% of deaths in LICs to 33% in upper middle-income countries.

### Deaths and YLLs Averted

If the injury mortality rates in LMICs were reduced to rates in HICs – through improvements in both injury prevention and trauma care – the number of injury-related deaths worldwide across all age groups would be 1,826,468, with 2,061,687 lives saved per year. This assumes that HICs would maintain their current rate regardless of whether they were above or below the average HIC rate (47.3 per 100,000 people). In individual HICs with mortality rates above the HIC average, reducing their rates to the HIC group average would save an additional 55,813 lives yearly, for a total of 2,117,500 lives saved per year. This represents 48% of annual injury-related deaths worldwide. The net DALYs averted, without discounting or age weighting, by reducing injury mortality in LMICs ranges from 102 million years to 103 million years, which is approximately 43% of current DALYs. With discounting and age weighting with a peak at 25 years (β = 0.04), the net DALYs averted ranges from 49 million years to 52 million years, which is between 33% and 36% of current DALYs (table 2).

**Table 2:** Net deaths and DALYs averted by income group.

Net deaths and YLLs averted by income group			
Income Group	Net deaths averted, in thousands(% of total)	Net DALYs [0,0,0] averted, in thousands(% of total)	Net DALYs [3,1,β] averted, in thousands(% of total)
Low	453 (71.1)	24,685–24,735* (66.9–67.1*)	12,205–12,557* (33.1–34.0*)
Lower Middle	1,039 (58.3)	52,482–52,838* (52.6–53.0*)	25,520–27,500* (25.6–27.6*)
Upper Middle	570 (39.2)	25,759–24,405* (32.3–34.1*)	11,428–12,176* (15.1–16.1*)
LMICs	2,062 (53.3)	102,926–101,978* (48.1*–48.5)	49,153–52,232* (23.2–24.6*)
High	56 (10.8)	2,413–2,638* (9.5–10.4*)	935–1,535* (3.7–6.1*)
World	2,118 (48.2)	105,339–104,617* (44.1*–44.4)	50,088–53,767* (21.1–22.7*)

Net deaths are deaths averted if injury mortality rates for each country were to match overall injury mortality rates in HICs (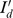). Net DALYs are reported without discounting or age weighting (*Net DALYs [*0,0,0]), and with discounting of 3% and age weighting with a peak at 25 years, or β = 0.04 (*NetDALYs* [3,1,β]). The ranges of reported estimates are from the results of methods 1 and 2 in calculating net DALYs (see Methods). Net DALY values without an asterisk represent DALYs averted if injury mortality rates for each country were to match overall injury mortality rates in HICs in each age group (method 1, 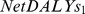). The values with an asterisk (*) represent DALYs averted if injury mortality and disability matched those of HICs method 2, 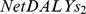).

### Economic Benefit

Using the human capital approach, estimates of the economic benefit, without discounting or age weighting, of reducing mortality rates in LMICs to the average rate in HICs range from 508 to 520 billion U.S. dollars, which is between 1.44% and 1.47% of the GNI of all LMICs. With discounting and age weighting to a peak age of 25 years (β = 0.04), economic benefit ranges from 245 to 261 billion U.S. dollars, which is between 0.69% and 0.74% of the GNI of all LMICs. With the VSL approach, the economic benefit of reducing injury mortality rates is between 758 and 786 billion U.S. dollars (table 3).

**Table 3:** Economic benefit of reducing injury mortality.

Total economic benefit by income group			
Income Group	Human capital approach, in USD billions (% of total GNI)		VSL approach, in USD billions
	[0,0,0]	[3,1,β]	[3,1, 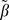 ]
Low	28.8–28.9 (3.09–3.10)	14.3–14.8 (1.53–1.58)	14.6–14.8
Lower Middle	181.5–183.1 (1.99–2.01)	88.6–96.3 (0.97–1.06)	169.0–174.0
Upper Middle	310.1–296.5 (1.17–1.23)	142.2–150.3 (0.56–0.60)	569.2–602.6
LMICs	520.4–508.5 (1.44–1.47)	245–261.4 (0.69–0.74)	758.0–786.3
High	95.5–94.2 (0.22–0.23)	39–50.3 (0.09–0.12)	329.2–348.6
World	616–602.7 (0.78–0.80)	284–311.7 (0.37–0.40)	1,106.6–1,115.5

Economic benefit is reported as a range. Economic benefit calculated by the human capital approach is reported without discounting or age weighting (0,0,0), and with discounting of 3% and age weighting to a peak at age 25 years, or β = 0.04 (3,1, β). The percentage of the total GNI for each income group is reported in parentheses below the range of estimates. Economic benefit calculated by the VSL approach is reported only with discounting of 3% and age weighting to a peak at two-thirds the standard life expectancy at birth, or β = 0.017 [3,1,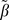].

## Discussion

We found that reduction in injury mortality rates in LMICs to the average HIC rate could save over 2.0 million lives per year worldwide. If HICs with mortality rates above the average also successfully reduced their rates, this would save another 55,813 lives, for a total of over 2.1 million lives saved annually. From an economic perspective, using discounted and age weighted values that we believe provide a more accurate assessment of economic benefit, this is worth between approximately 250 billion dollars (using the human capital approach) and approximately 760 billion dollars (using the VSL approach).

Historically, global health efforts have focused on infectious and communicable diseases, including HIV/AIDS, tuberculosis and malaria. The widespread nature of these diseases, coupled with transmissibility, the presence of treatments viewed as cost-effective, and strong advocacy from health care professionals and patients strengthened efforts to prioritize treatment of these diseases in resource-limited settings. Surgical care has classically not been thought to be in the purview of global health, in part because of questions regarding the cost-effectiveness and feasibility of surgical interventions. Surgical care was so noticeably absent from global health discourse that surgery was deemed “the neglected stepchild of global health” [23].

More recently, however, surgical interventions have been proven extremely cost-effective, including cataract repair [24], caesarean section for obstructed labor [10] and circumcision [25]. Likewise, many of the interventions needed to improve trauma care, such as improving pre-hospital capabilities and strengthening surgical capacity at first level hospitals, are among the most cost-effective of all health care interventions [26]. This study provides additional evidence that investment in surgical services – a crucial component of trauma care – in addition to continued work on injury prevention, can provide substantial economic benefit in resource-limited settings. Injury disproportionately affects younger, more productive members of society, heightening the impact of averting injury deaths. Previous efforts to improve injury-related outcomes in resource-limited settings have including pre-hospital initiatives training commercial drivers in first responder principles in Ghana [27]. Further such efforts, as well as initiatives focused on trauma care training, infrastructure development, and resource procurement, will be necessary in resource-limited settings to reduce injury morbidity and mortality.

### Limitations

While this study provides substantial evidence of the economic benefit of reduced injury mortality, these findings must be interpreted in the context of the study’s limitations. First, this study makes necessary assumptions regarding the demographics of those affected by injury and the relationship between injury mortality and disability. For *method 1*, we assumed that even with reduced mortality rates, life expectancy, average age of death, and YLDs would remain the same in LMICs. We attempted to adjust for this in *method 2*, with these factors matching HICs. Both methods represent a theoretical scenario that is unrealistic, one where nothing in LMICs has changed except mortality rates, and the other where everything has changed to match HICs. However, we present these results as a range of values possible if better trauma care, and injury prevention interventions, were provided in LMICs. Additionally, it is important to note that all individuals within a country are grouped together into a country income group based on per capita income, regardless of any one individual’s personal income. Secondly, it is unlikely that injury mortality rates equivalent to those in HICs can be reached in LMICs without significant global investment. The estimates of economic benefit therefore remain a long-term vision. Nevertheless, given the magnitude of potential benefit, even small improvements in trauma care capabilities could have significant effects, as mortality reductions of 8–10% have been shown from improvements in trauma system development. [28]–[30] Secondly, due to the limitations of data from the 2010 GBD study, our current calculations reflect both injury rates and mortality from these injures, without isolation of these components. Ideally, we would have calculated the rate of injury in the population (injuries per 100,000 people) and the mortality per injury (deaths per 100,000 injuries) separately. By holding the injury rate constant for LMICs, and changing the mortality rate only, this would have given a clearer picture of the effect of improved trauma care on reducing mortality, without changing the incidence of injury itself. Instead we had to calculate mortality rates by population, which are affected by trauma care as well as by injury prevention. Our results therefore represent the benefit of reductions in mortality from improvements in both injury prevention and trauma care. Third, the human capital approach used in this analysis has weaknesses (described above), most notably that the value of an individual life is based on earning potential which varies by setting and is not (or should not be) a reflection of whether that life is worth saving from a medical perspective. For this reason, the VSL approach may be favorable since it describes an individual’s willingness to pay for actions that reduce their risk of death. The VSL can therefore exceed lifetime earnings, and can be 1–2 orders of magnitude higher than human capital estimates, as seen in this study [9]. Fourth, this study focuses on strictly a measure of economic gain resulting from improved injury prevention and trauma care, and does not attempt to complete a full cost benefit analysis, taking into account costs such as unemployment rates, costs of treatment, etc. Such an analysis would be exceedingly difficult to perform globally without detailed information on health infrastructure and economics of each country, and the demographics of each patient affected by trauma, and was outside of the scope of this project.

In conclusion, this study demonstrates the vast numbers of lives that could be saved by reductions in injury mortality rates in LMICs to the average rate in HICs, and the substantial associated economic benefit. Significant efforts have been made in recent years to improve surgical infrastructure, develop surgical training programs, and expand surgical services in resource-limited settings, to improve trauma care overall. Further investments in these arenas, as well as continued work on injury prevention, have the potential to save millions of lives and provide significant economic benefit globally.
